# Novel Insights into Young Adults’ Perceived Effectiveness of Waterpipe Tobacco-Specific Pictorial Health Warning Labels in Lebanon: Implications for Tobacco Control Policy

**DOI:** 10.3390/ijerph18137189

**Published:** 2021-07-05

**Authors:** Rima Nakkash, Malak Tleis, Sara Chehab, Wu Wensong, Michael Schmidt, Kenneth D. Ward, Wasim Maziak, Taghrid Asfar

**Affiliations:** 1 Department of Health Promotion and Community Health, Faculty of Health Sciences, American University of Beirut, Beirut 1103, Lebanon; rn06@aub.edu.lb (R.N.); mt99@aub.edu.lb (M.T.); sc25@aub.edu.lb (S.C.); 2 Department of Mathematics and Statistics, Florida International University, Miami, FL 33199, USA; wenswu@fiu.edu; 3 Department of Art, University of Memphis, Memphis, TN 38152, USA; mschmidt@memphis.edu; 4 School of Public Health, University of Memphis, Memphis, TN 38152, USA; kdward@memphis.edu; 5 Department of Epidemiology, Robert Stempel College of Public Health and Social Work, Florida International University, Miami, FL 33199, USA; wmaziak@fiu.edu; 6 Syrian Center for Tobacco Studies, Aleppo 2203, Syria; 7 Sylvester Comprehensive Cancer Center, University of Miami Miller School of Medicine, Miami, FL 33124, USA; 8 Department of Public Health Sciences, University of Miami, Miller School of Medicine, Miami, FL 33124, USA

**Keywords:** waterpipe, health warning labels, hookah

## Abstract

This study aims to explore the perceived effectiveness of waterpipe (WP) tobacco specific health warning labels (HWLs) among young adult WP smokers and nonsmokers in Lebanon. Before participating in focus group discussions, participants (*n* = 66; WP smokers *n* = 30; nonsmokers *n* = 36; age 18–33) completed a brief survey to rate the effectiveness of 12 HWLs’ and rank them according to four risk themes (WP health effects, WP harm to others, WP-specific harm, and WP harm compared to cigarettes). Differences in HWLs ratings by WP smoking status were examined and the top-ranked HWL in each theme were identified. HWLs depicting mouth cancer and harm to babies were rated as the most effective by both WP smokers and non-smokers. WP smokers rated HWLs which depicted harm to children and infants as more effective than non-smokers. The top-ranked HWLs for perceived overall effectiveness were those depicting “oral cancer”, “harm to babies”, “orally transmitted diseases” and “mouth cancer”. HWLs depicting oral lesions and harm to babies were rated as most effective, while HWLs showing the harmful effects of WP secondhand smoke on infants and children were rated as less effective by nonsmokers compared to smokers. Our study provides evidence on the potential effectiveness of HWLs for further evaluation in Lebanon and the Eastern Mediterranean region. The results will inform and guide the development and implementation of tobacco control policy.

## 1. Introduction

Waterpipe (WP) smoking continues to increase globally, and more so in the Eastern Mediterranean Region (EMR) [1,2]. As in most countries in the region, WP smoking has become the number one tobacco use method among young adults in Lebanon [3]. Among a sample of 1680 adults (50% females; 64% less than 45 years), WP smoking in Lebanon was 39.5% in 2019, three-times higher than in Jordan and Palestine (11% and 12.9% respectively) [4]. As for youth, in a review of WP prevalence and trends, current use was the highest among Lebanese youth (37.2% in 2008), and having ever used was the highest among Lebanese youth in 2002 and Lebanese university students in 2005 (both 65.3%) [3]. A more recent study of 3384 students from 17 universities in Lebanon (2014), showed that 23% were current WP smokers compared to 19.2% for cigarettes [5]. Additionally, 5–6% of Lebanese expectant mothers reported WP smoking during pregnancy [6].

One of the main drivers of the WP epidemic has been the widespread misperception of its relative safety compared to cigarettes [7]. Evidence suggests WP smoking carries similar health risks as cigarettes. Moreover, unique risks of WP can stem from the contribution of charcoal (e.g., carcinogens), device and accessories (e.g., infectious disease) [8]. While this perception runs against evidence of considerable risks [9], it is indicative of the important gap in communicating the harmful and addictive nature of WP smoking with young people [10].

Health warning labels (HWLs) have been one of the main strategies to communicate smoking-related health risks [11] and were proven effective in encouraging cigarette smoking cessation and discouraging initiation among young people [12]. They also serve as constant sources of health information for nonsmokers, being displayed each time the product is used [13]. Pictorial labels, mainly graphic ones, were found to reduce motivation to smoke [14] and influence attitude towards smoking [15] among youth. In Lebanon, evaluation of pictorial HWLs on cigarettes showed higher effectiveness than in message-related and impact-related variables, including intentions to quit or not to start smoking among high school and university students [16]. Using pictorial HWLs on tobacco products can offer a promising policy to curb the WP tobacco smoking epidemic [17]. Moreover, complying with article 11 of the Framework Convention on Tobacco Control (FCTC) entails the use of suitable pictorial HWLs on all tobacco products, including waterpipes [18].

In 2005, Lebanon ratified the FCTC, but it was not until 2011 that a comprehensive tobacco control law was passed by Parliament. The passage of the law followed a two year advocacy campaign which pushed for a tobacco control policy in line with the FCTC [19]. Lebanese Law 174 includes an article that requires the use of larger textual health warning labels and eventually pictorial warnings labels. Although a decree (8991 Article 2 in Law 174) requiring larger textual health warnings was issued on, to date there has been no progress on moving forward a decree requiring pictorial health warnings.

Recently, our team led the development of a set of 12 HWLs, corresponding to four themes; WP health effects, WP harm to others, WP-specific harm, and WP harm compared to cigarettes through a Delphi study [20]. First, the team identified priority themes from the literature, reviewed existing cigarette HWLs, and finalized the content and main design parameters for the pictorial HWLs, resulting in 28 WP-specific HWLs. Subsequently, a three-round Delphi study was conducted with an international expert panel to reach a consensus on a set of the most effective HWLs for each theme [20]. To further optimize and adapt the HWLs to the local context and population in Lebanon, a formative evaluation using focus groups (FG) was conducted among young adults [13,21]. As part of the FG, we conducted a brief individual survey to (1) examine differences in HWLs ratings on four outcomes (effectiveness concerning thinking about WP health risks; thinking about quitting; preventing WP smoking initiation; and perceived overall effectiveness of HWL) according to WP smoking status (smokers vs. non-smokers), and (2) identify the top-ranked HWL within each theme. Information from the FG discussions will guide the adaptation and improvements of HWLs, while analysis of the rating and ranking will provide a snapshot of how HWLs are perceived by the target population on several communication outcomes. We report here results of the rating and ranking to help prioritize HWLs with the most potential for use in the EMR and advance evidence and research related to the implementation of WP HWLs. The ultimate goal is to assist Lebanon in adapting and improving HWL implementation, as part of advancing its tobacco control policy. This research will provide the Lebanese government with an array of potential waterpipe-specific pictorial HWLs to include in the regulatory decree that needs to be issued as per Law 174.

## 2. Materials and Methods

The study was approved by the Institutional Review Board at the American University of Beirut on 17 September 2020 (SBS-2020-0267) and the institutional review board at Florida International University on 26 November 2018 (IRB-17-0134-AM03). Nine mixed-gender FGs combined with a brief survey (four FGs with WP smokers; five FGs with nonsmokers) were conducted from February to July 2019 among young adults (*n* = 66; 30 smokers, 36 nonsmokers; 53% females; age 18–33 years) in Beirut, Lebanon. To recruit participants, flyers were distributed around four major universities in Beirut, aimed towards young adults 18–34 years of age. We included both WP smokers and nonsmokers, where WP smokers were defined as those who smoked WP even once in the last 30 days. In addition, a list of potential participants, who participated in previous research and consented to be re-contacted for future research, was provided by a local recruitment company as potential participants for the FG discussion. Interested participants were contacted and screened for eligibility by phone and were scheduled for an FG session which were held in a meeting room at the American University of Beirut. Before the FG discussion, two public health researchers explained the study, consented participants, and discussed the content and guidelines for participating in the FGs. Then, participants completed a survey including (1) socio-demographics (i.e., age, sex, education, and employment), (2) smoking behavior (frequency of smoking: cigarettes, WP and other tobacco products) and (3) ratings of HWLs and (4) ranking of the HWLs within each of the 4 themes. For the last part, participants were asked to view each HWL and then rate its effectiveness in (1) prompting thinking about health risks of WP smoking, (2) motivating smokers to quit WP or think about quitting (3) preventing young adults from initiating WP smoking, and (4) perceived overall effectiveness of the HWL, i.e., how much the participant anticipated the HWL could influence their perception of WP smoking harm, or prompt them to think about changing their using habits. All four outcomes were measured on a 4 point scale (from 1 = not at all effective to 4 = very effective). Ranking was done per theme where participants arranged HWLs from least to most effective in each theme in terms of overall perceived effectiveness.

For the rating of HWLs, participants were asked to view each HWL and then rate its effectiveness on a scale from 1 (not at all effective) to 4 (very effective). Effectiveness was evaluated by four factors, i.e., effectiveness of the HWL in: (1) prompting thinking about WP health risks; (2) prompting thinking about quitting; (3) preventing WP smoking initiation; and (4) perceived overall effectiveness of HWL. For ranking, participants were asked to arrange HWLs in each theme from most to least effective based on overall effectiveness, adapted from literature [22,23]. For the evaluation of HWLs, we adopted the message impact framework that is based on communication and psychological theories [24,25,26] and summarizes the approaches used commonly in tobacco warnings research [23]. Rating and ranking were done within themes to be able to select HWLs for future testing in an experimental study that addresses the various themes.

Non-parametric tests were used to test differences in HWLs rating by WP smoking status. Freidman’s test (*p* < 0.05) was then used to compare the distributions of ratings (within-subject effect) of HWLs in each theme. Wilcoxon signed-rank test post hoc was used to examine the pairwise difference between HWLs effectiveness in each theme, with a Bonferroni correction applied for multiple comparisons (at *p* < 0.05) [27]. Mann–Whitney U test (U) was used to examine differences in rating distributions of HWLs effectiveness between WP smokers and nonsmokers. For HWLs ranking, a simple descriptive analysis of proportion was used to reflect ranking in terms of overall HWLs effectiveness in each theme. Statistical software SPSS version 25 was used [28].

## 3. Results

### 3.1. Sample Characteristics

Of the 66 participants in the focus group discussions (53% females; mean age (SD) = 23.77 (3.71) y), 45% were WP smokers and 55% were WP nonsmokers overall, 50% of the participants held undergraduate degrees, 39% were current university students, and 50% were employed, as shown in Table 1. Among WP smokers, 54% initiated smoking between the ages of 14 and 18 years, and 46% considered themselves as not addicted to WP smoking, while 19% labeled themselves as very addicted to WP smoking. The number of WPs smoked per month was 1–5 for 38% of participants.

### 3.2. HWLs Ratings

The mean effectiveness ratings for each warning label are shown for WP smokers and nonsmokers, and between HWLs within the same theme in Table 2.

#### 3.2.1. Differences in Rating HWLs in Each Theme

In theme 1 “WP health effect”, WP smokers significantly rated HWL#1 “WP cause oral cancer” higher than HWL#3 “facial wrinkles” (*p* = 0.002) for overall effectiveness. Similar ratings were given for the HWLs in theme 3 “WP specific harms” by WP smokers and nonsmokers. Both groups significantly rated HWL#7 “sharing WP transmits infectious diseases” as more effective than HWL#9 “WP toxins are not filtered by water” for overall effectiveness (*p* = 0.002; *p* < 0.0001, respectively). Additionally, WP smokers and nonsmokers rated HWL#8 “WP smoking spreads infectious diseases” as more effective than HWL#9 for overall effectiveness (*p* < 0.0001; *p* = 0.002, respectively).

#### 3.2.2. WP Smokers vs. WP Nonsmokers—Rating Specific Outcomes

When compared according to the three specific communication outcomes: thinking about WP health risks, thinking about quitting, and preventing WP smoking initiation, differences between WP smokers and nonsmokers were detected in themes 2 and 3, as shown in Table 3.

In theme 2 “harm to others”, WP smokers rated HWL#4 “harm to babies” (*p* = 0.008), HWL#5 “harm to children” (*p* < 0.0001), and HWL#6 “harm to fetuses” (*p* = 0.002) as more effective in preventing youth from initiating WP smoking, in comparison to WP nonsmokers. Additionally, WP smokers found HWL#4 “harm to babies” (*p* = 0.037) and HWL#6 “harm to fetuses” (*p* = 0.031) more effective in encouraging quitting WP than WP nonsmokers. While for theme 3 “WP Specific Harms”, compared to WP nonsmokers, smokers significantly rated HWL#8 “WP smoking spreads infectious diseases” as more effective in preventing youth from initiating WP smoking (*p* = 0.031).

#### 3.2.3. WP Smokers vs. WP nonsmokers—Rating Total Effectiveness

The differences in rating the total effectiveness of the HWLs are shown in Table 3. Differences emerged between ratings of HWLs between WP smokers and nonsmokers in Theme 2 “harm to others”. WP smokers significantly rated all HWLs higher on overall effectiveness than nonsmokers. While for theme 4 “WP harm compared to cigarettes”, only WP nonsmokers rated HWL#10 “WP causes oral cancer, as cigarettes” better than HWL#11 “heart disease” (*p* = 0.012) and HWL#12 “WP smokers inhale 100 times more smoke than cigarettes smokers” (*p* = 0.01) in overall effectiveness. WP smokers rated HWL#10 significantly higher than HWL#12 (*p* = 0.005) in overall effectiveness.

#### 3.2.4. HWLs ranking

The top-ranked HWLs for perceived overall effectiveness per theme were: 1) HWL#1 “WP cause oral cancer” (42.9%) in theme 1 “WP health effects”; 2) HWL#4 “harm to babies” (55.8%) in theme 2 “harm to others”; 3) HWL#7 “sharing WP transmits infectious diseases” (64.9%); in theme 3 “WP specific harms”; and 4) HWL#10 “WP causes oral cancer, as cigarettes” (58.4%) in theme 4 “WP harm compared to cigarettes” (Table 3). Similar rankings were shown for WP smokers and nonsmokers.

## 4. Discussion

This study is the first to report on the assessment of WP-specific HWLs on: thinking about WP health risks; thinking about quitting; preventing WP smoking initiation, and perceived overall effectiveness of HWL among young adults in Lebanon.

In detail, the proposed HWLs depicting mouth cancer and harm to babies were rated as the most effective by WP smokers and non-smokers. WP smokers tended to rate HWLs showing WP harmful effects on others higher than non-smokers. HWLs with images of mouth sores received the highest ratings, regardless of smoking status. Our results are consistent with prior research suggesting that HWLs on tobacco products with pictorials depicting diseases are more likely to promote smoking cessation or evoke intentions to quit [13] in the EMR [29] and globally [30]. As the drivers of the WP epidemic in the EMR share similar traits on both the individual and cultural levels [31], our results can guide further adaptation and implementation of WP HWLs in EMR and has valuable implications on informing policy development

When stratified by WP smoking status, differences appear in the theme of “harm to others,” where all three HWLs depicting harm to fetuses, babies, and children from exposure to WP secondhand smoke received higher rating among WP smokers than non-smokers. Similar results were reported in the US and Jordan [32,33]. This result might be due to greater concern among young adult WP smokers of reproductive age for the consequences of smoking on infants and children, compared to their health [32]. In addition, the differences detected in rating this set of HWLs between WP smokers and nonsmokers indicate that adopting WP-specific HWLs policy in Lebanon has the potential to address both initiation of WP and reduction/cessation of WP smoking.

Limitations to consider when interpreting our results mainly relate to the small sample size and convenience sampling. Moreover, we assess in this manuscript the perceived effectiveness of the HWL rather than the actual effectiveness. Additionally, we compare the rating of HWLs within themes, preventing us from knowing the best HWLs among all. Nonetheless, this allowed us to recommend a group of the best-rated HWLs across several themes.

## 5. Conclusions

Lebanon suffers from considerable waterpipe (WP) smoking among young adults. Although cigarette specific health warning labels have been evaluated in the Lebanese context [16], to date this has not extended to waterpipe-specific HWLS. Globally, the development and testing of WP-specific HWLs have been consistently identified as a priority for WP control by leading public health and tobacco control organizations [34], but to date there have been no systematic efforts to develop and test HWLs. We examined differences in HWLs ratings and ranking on: effectiveness concerning thinking about WP health risks; thinking about quitting; preventing WP smoking initiation; and perceived overall effectiveness of HWL, by WP smoking status among young adults in Lebanon.

HWLs that are most promising to use for the general population are the ones depicting mouth cancer and harm to babies. Moreover, the difference detected in rating HWLs between WP smokers and nonsmokers emphasis the role of HWLs in addressing initiation and cessation. A policy recommendation to consider is to rotate the HWLs that will be used on the WP components, with higher use of HWLs more relevant to WP smokers, as per FCTC guidelines in implementing HWLs [12].

Given the seriousness of the WP epidemic in the EMR and the scarcity of research on WP HWLs, we provide timely evidence of the potential effectiveness of WP-specific HWLs as well as recommendations of potential warnings to adopt in policy that are tailored to WP smoking status. Future research directions involve testing the most effective HWLs in an online experimental study to measure their effectiveness when applied on the various WP components including the tobacco package and other WP accessories.

## Figures and Tables

**Table 1:** Demographic characteristics and smoking status of focus group discussion participants (*n* = 66).

Variable	N (%)
Gender	
Females	35 (53)
Males	29 (44)
Age (Mean [SD])	23.8 [3.71]
Education	
Undergraduate degree/Bachelor’s degree	33 (50)
Graduate degree/Master’s degree	15 (23)
Technical school	2 (3)
University student (yes)	26 (39)
Employed (yes)	33 (50)
Current cigarette smoker (yes)	11 (17)
Current WP smoker (yes)	30 (45)
Age of WP initiation (only WP smokers)	
<13	3 (12)
14–18	14 (54)
19–25	9 (35)
Hooked on WP (only WP smokers)	
Not hooked	12 (46)
Somehow hooked	9 (35)
Very hooked	5 (19)
Average number of WP smoked per month (only WP smokers)	
1–5	10 (38)
10–25	9 (35)
≥30	5 (19)
Harm perception of WP compared to cigarettes (only WP smokers)	
Less harmful	4 (15)
Equally harmful	6 (23)
More harmful	12 (46)
Don’t know	2 (8)
Intention to quit WP smoking in future	
Within next month	1 (4)
Within next 6 month	3 (12)
Beyond 6 month	7 (27)
Not planning to quit	12 (46)
Tried to quit before (yes)	11 (42)

Differences in percentages account for missing data. Percentages are rounded to the highest whole digit. Current WP and cigarette smokers are those who declared daily, weekly and every few weeks use. Current employee included full time, part-time and self-employed.

**Table 2:** Health warning labels rating (mean) and rankings (%) by themes and by smoking status (all, WP smokers, WP non-smokers).

Labels		Overall Effectiveness			Ranking (%)
		All	WP Smokers	WP Nonsmokers	All
Theme 1: WP Health Effects					
HWL1	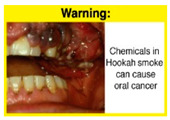	3.18 ^a^	3.27 ^a^	3.08 ^a^	42.9
HWL2	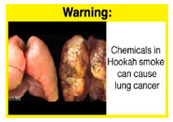	3.07 ^a,b^	3.13 ^a,b^	2.94 ^a,b^	31.3
HWL3	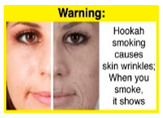	2.61 ^c^	2.67 ^b^	2.64 ^a,b^	9.1
Theme 2: Harm to Others					
HWL4	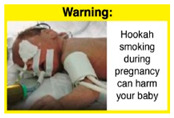	3.4 ^a^	3.64 ^a^	3.25 ^a^	55.8
HWL5	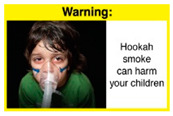	3.11 ^b^	3.39 ^a^	2.92 ^a^	20.8
HWL6	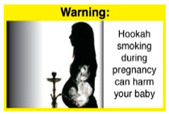	3.07 ^b^	3.48 ^a^	2.74 ^a^	18.2
Theme 3: WP Specific Harms					
HWL7	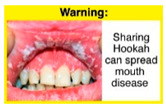	2.97 ^a^	3 ^a^	3 ^a^	64.9
HWL8	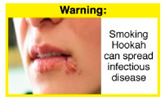	2.91 ^a^	3 ^a^	2.78 ^a^	20.8
HWL9	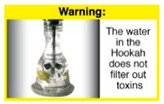	2.07 ^b^	2.13 ^b^	2 ^b^	7.8
Theme 4: Comparison to Cigarette					
HWL10	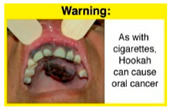	3.24 ^a^	3.3 ^a^	3.37 ^a^	58.4
HWL11	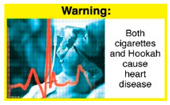	2.87 ^b^	2.97 ^a,b^	2.94 ^b^	18.2
HWL12	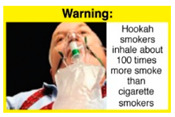	2.68 ^b^	2.81 ^b^	2.71 ^b^	15.6

Different superscript letters ^a,b,c^ denote significant differences for HWLs pairwise comparisons within the same theme, using Wilcoxon signed-rank test post hoc with a Bonferroni correction (*p* < 0.0125 for theme 1 and *p* < 0.017 for themes 2, 3 and 4). Warnings within same set with same superscript letter are not significantly different from one another. Bolded and underlined scores denote a significant difference (at *p* < 0.05) between WP smokers and nonsmokers for that particular HWL, using Mann–Whitney U tests. Ratings of HWLs is reflected by mean of the effectiveness values, measured on a 4 point scale and “% ranked as most effective” presents the number of times the HWL was chosen as the most effective in its corresponding theme. Percentages do not sum up to 100 due to missing values.

**Table 3:** Health warning labels rating (mean) and rankings (%) by themes and by smoking status (All, WP smokers, WP non-smokers) for the three specific outcomes.

Labels		Thinking of WP Health Risks			Thinking about Quitting WP			Preventing from Starting WP		
		All	Smokers	Nonsmokers	All	Smokers	Nonsmokers	All	Smokers	Nonsmokers
Theme 1: WP Health Effects										
HWL1	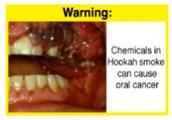	3.12 ^a^	3.23 ^a^	3.28 ^a^	2.99 ^a^	3.03 ^a^	2.94 ^a^	3.21 ^a^	3.23 ^a^	3.14 ^a^
HWL2	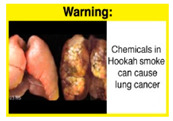	2.97 ^a,b^	2.80 ^a,b^	3.06 ^a,b^	2.92 ^a,b^	2.97 ^a^	2.86 ^a^	2.95 ^b^	2.93 ^a^	2.89 ^a,b^
HWL3	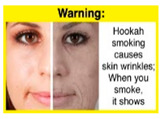	2.67 ^b^	2.63 ^b^	2.64 ^b^	2.48 ^c^	2.57 ^a^	2.47 ^a^	2.63 ^b^	2.73 ^a^	2.53 ^b^
Theme 2: Harm to Others										
HWL4	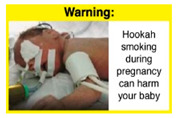	3.50 ^a^	3.64 ^a^	3.39 ^a^	3.38 ^a^	3.57 ^a^	3.19 ^a^	2.97 ^a^	3.32 ^a^	2.64 ^a^
HWL5	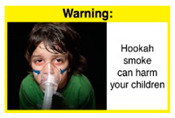	3.27 ^a,b^	3.21 ^a^	3.19 ^a^	3.07 ^a,b^	3.25 ^a^	2.94 ^a^	2.67 ^b^	3.21 ^a^	2.17 ^b^
HWL6	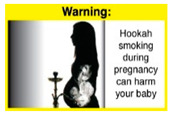	3.20 ^b^	3.33 ^a^	3.03 ^a^	3.08 ^b^	3.34 ^a^	2.80 ^a^	2.73 ^a,b^	3.17 ^a^	2.34 ^a,b^
Theme 3: WP Specific Harms										
HWL7	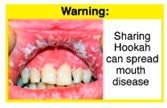	3.11 ^a^	3.13 ^a^	3.08 ^a^	3.00 ^a^	3.07 ^a^	2.92 ^a^	2.95 ^a^	2.97 ^a^	3.00 ^a^
HWL8	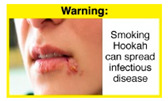	2.84 ^b^	3.10 ^a^	2.72 ^b^	2.87 ^a^	3.00 ^a^	2.75 ^a^	2.68 ^b^	2.93 ^a^	2.44 ^b^
HWL9	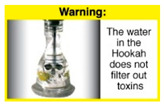	2.13 ^c^	2.16 ^b^	2.12 ^c^	2.12 ^b^	2.13 ^b^	2.12 ^b^	2.08 ^c^	2.10 ^b^	2.09 ^b^
Theme 4: WP harm Compared to Cigarette										
HWL10	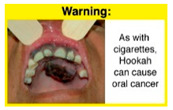	3.20 ^a^	3.00 ^a^	3.23 ^a^	3.27 ^a^	3.30 ^a^	3.29 ^a^	3.12 ^a^	3.30 ^a^	3.23 ^a^
HWL11	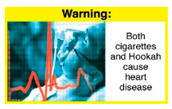	2.93 ^b^	2.97 ^a^	2.94 ^a,b^	2.89 ^b^	2.97 ^a,b^	2.89 ^a,b^	2.75 ^b^	2.87 ^a,b^	2.71 ^b^
HWL12	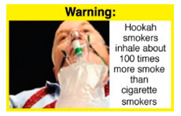	2.78 ^b^	2.81 ^a^	2.71 ^b^	2.75 ^b^	2.71 ^b^	2.77 ^b^	2.64 ^b^	2.65 ^b^	2.71 ^a,b^

Different superscript letters ^a,b,c^ denote significant differences for HWLs pairwise comparisons within the same theme, using Wilcoxon signed-rank test post hoc with a Bonferroni correction (*p* < 0.0125 for theme 1 and p < 0.017 for themes 2, 3 and 4). Warnings within same set with same superscript letter are not significantly different from one another. Bolded and underlined scores denote a significant difference (at *p* < 0.05) between WP smokers and nonsmokers for that particular HWL, using Mann–Whitney U tests. Ratings of HWLs is reflected by mean of the effectiveness values, measured on a 4 point scale and “% ranked as most effective” presents the number of times the HWL was chosen as the most effective in its corresponding theme. Percentages do not sum up to 100 due to missing values.

## Data Availability

Data is contained within the article or supplementary material.
